# The combined induction of liver progenitor cells and the suppression of stellate cells by small molecules reverts chronic hepatic dysfunction

**DOI:** 10.7150/thno.54457

**Published:** 2021-03-14

**Authors:** Wei-Jian Huang, Xu Zhou, Gong-Bo Fu, Min Ding, Hong-Ping Wu, Min Zeng, Hong-Dan Zhang, Ling-Yan Xu, Yi Gao, Hong-Yang Wang, He-Xin Yan

**Affiliations:** 1 International Cooperation Laboratory on Signal Transduction, Eastern Hepatobiliary Surgery Hospital, Second Military Medical University, Shanghai, China.; 2 Department of Hepatobiliary Surgery II, Guangdong Provincial Research Center for Artificial Organ and Tissue Engineering, Zhujiang Hospital, Southern Medical University.; 3 Department of Medical Oncology, Jinling Hospital, First School of Clinical Medicine, Southern Medical University.; 4 Department of Interventional Oncology, Renji Hospital, Jiaotong University School of Medicine, Shanghai, China.; 5 Shanghai Celliver Biotechnology Co. Ltd., Shanghai, China.; 6 Department of Pharmacy, Shanghai Jiao Tong University Affiliated Sixth People's Hospital, East Campus, Shanghai, China.; 7 Shanghai Cancer Institute, Renji Hospital, Shanghai Jiaotong University School of Medicine, Shanghai, China.

**Keywords:** small molecules, *in vivo* reprograming, liver fibrosis, liver progenitor cells, stellate cells

## Abstract

**Rationale:** We developed a cocktail of soluble molecules mimicking the *in vivo* milieu supporting liver regeneration that could convert mature hepatocytes to expandable liver progenitor-like cells *in vitro*. This study aimed to induce endogenous liver progenitor cells by the administration of the soluble molecules to provide an alternative approach for the resolution of liver fibrosis.

**Methods:** *In vitro* cultured hepatocyte-derived liver progenitor-like cells (HepLPCs) were transplanted into CCL4-treated mice to investigate the therapeutic effect against liver fibrosis. Next, we used HGF in combination with a cocktail of small molecules (Y-27632, A-83-01, and CHIR99021 (HACY)) to induce endogenous CD24^+^ liver progenitor cells and to inhibit the activation of hepatic stellate cells (HSCs) during CCL4-induced hepatic injury. RNA sequencing was performed to further clarify the features of HACY-induced CD24^+^ cells compared with CCL4-induced CD24^+^ cells and *in vitro* derived HepLPCs. Finally, we evaluated the expansion of HACY-induced CD24^+^ cells in human hepatocyte-spheroids from fibrotic liver tissues.

**Results:** HepLPCs exhibited the capacity to alleviate liver fibrosis after transplantation into CCL4-treated mice. The *in vivo* administration of HACY not only induced the conversion of mature hepatocytes (MHs) to CD24^+^ progenitor cells but prevented the activation of HSCs, thus leading to enhanced improvement of liver fibrosis in CCL4-treated mice. Compared to CD24^+^ cells induced by CCL4 alone, HACY-induced CD24^+^ cells retained an enhanced level of hepatic function and could promote the restoration of liver function that exhibited comparable gene expression profiles with HepLPCs. CD24^+^ cells were also observed in human liver fibrotic tissues and were expanded in three-dimensional (3D) hepatic spheroids in the presence of HACY *in vitro*.

**Conclusions:** Hepatocyte-derived liver progenitor-like cells are crucial for liver regeneration during chronic hepatic injuries. The administration of HACY, which allowed the induction of endogenous CD24^+^ progenitor cells and the inactivation of HSCs, exerts beneficial effects in the treatment of liver fibrosis by re-establishing a balance favoring liver regeneration while preventing fibrotic responses.

## Introduction

The liver is a vital organ for homeostasis with high regenerative potential in terms of recovery of mass and function after injury 1. A variety of factors can cause damage to the liver, including viruses, alcohol use, and obesity. Typically, these factors impair hepatic regeneration and elicit fibrotic responses, resulting in chronic liver dysfunction 2. Despite the fact that hepatic stellate cells (HSCs) play a pivotal role in liver fibrosis, mature hepatocytes (MHs) are dominant cell type residing in the liver and their damage is commonly recognized as the key initiator of fibrosis by releasing pro-inflammatory factors to activate HSCs 3.

Apart for liver transplantation, there is no clinically effective therapy that has been approved for the treatment of fibrotic disease in the liver. Researchers have therefore been exploring new approaches to promote liver regeneration and revert fibrosis 4-6. As epithelial progenitor cells are thought to compensate for tissue loss in many adult tissues 7, stem/progenitor cell transplantation therapy has been considered as a promising alternative strategy. Notably, it has been shown that the transplantation of CD24^+^CD133^+^EpCAM^+^ liver progenitor cells, isolated from damaged liver, resulted in the repopulation of the hepatocellular parenchyma and a reduction of liver scarring 8. In addition, the transplantation of Lgr5^+^ cells has been shown to attenuate liver fibrosis and thus represents a valuable target for the treatment of liver damage 9. These findings indicated that progenitor cells may represent a continuous/responsive supply of resources to replenish the parenchyma and provide a diverse array of antifibrotic effectors for chronic liver injury and the restoration of liver function 10.

Previously, we developed a transition and expansion medium (TEM) that could culture and expand HepLPCs *in vitro* 11, 12. In this study, we reported that the transplantation of HepLPCs, with high levels of CD24 expression, effectively attenuated CCL4-induced liver fibrosis. Furthermore, given the abundant preclinical data relating to multiple small-molecule based approaches for the treatment of fibrosis, the direct administration of HACY including HGF, A-83-01(TGFβ-i), CHIR99021 (GSK3-i) and Y-27632 (ROCK-i), has been shown to give rise to the expansion of CD24^+^ progenitor cells and lead to the inactivation of HSCs in CCL4-treated mice* in vivo*, thus resulting in significant liver recovery in CCL4-treated mice. In addition, compared to CCL4-induced CD24^+^ cells, these HACY-induced CD24^+^ liver progenitor cells exhibited an analogous expression profile with HepLPCs cultured *in vitro*. These results indicate that the injection of a cocktail of HACY might be a valuable and applicable therapy for treating chronic liver fibrotic pathologies, both in mice and humans.

## Materials & methods

### Cell and spheroid culture

After isolation from the livers of mice, mature hepatocytes (MHs) were cultured in TEM and gradually converted into HepLPCs, as described previously 11. In brief, TEM was based on Advanced DMEM/F12 (Invitrogen) supplemented with N2 or insulin-transferrin-serine (ITS) (Invitrogen), and the following growth factors or small molecules: 20 ng/mL EGF, 20 ng/mL HGF (All Peprotech), 10 μM Y27632, 3 μM CHIR99021, 1 μM S1P, 5 μM LPA and 1 μM A83-01 (All MedChemExpress). TEM was changed every day. Primary HSCs were cultured in high-glucose Dulbecco's modified Eagle's medium containing 10% fetal bovine serum (FBS) and 1% penicillin/streptomycin with or without the same ratio of HACY in TEM. For the culture of three-dimensional (3D) spheroids, 1×10^6^ MHs were seeded in low-attachment 6-well plates with gentle shaking to form 3D spheroids, as described previously 13. Cells began aggregating within 6 h and formed cellular spheres after 48 h in HMM 14; these were cultured further in the presence or absence of HACY in HMM.

### Mouse experiments

Procedures involving mice were approved by the Institutional Animal Care and Use Committee at the Second Military Medical University. C57BL/6 J male mice (8 weeks of age; weighing approximately 20 g) were obtained from the Institute of Laboratory Animal Sciences, CAMS & PUMC (Beijing, China). For the induction of hepatic fibrosis by CCL4, the mice were injected (via the intra-peritoneal route) twice a week for 6 weeks with CCL4 (2 mL/kg, Sigma-Aldrich) dissolved in olive oil at a ratio of 1:4.

### Lentivirus and AAV infection

A lentivirus vector was created to carry GFP and a puromycin resistance gene and transfected into HepLPCs. After 48 h of puromycin treatment to eliminate uninfected cells, the expression of GFP was monitored by fluorescence imaging or FACS analysis.

For the *in vivo* tracking of mouse MHs, we created an AAV8-TBG-Cre construct containing the hepatocyte-specific thyroxin-binding globulin (TBG) promoter (Celliver Biotechnology Inc., Shanghai, China). This was administered intravenously at a concentration of 2×10^11^ plaque-forming units (pfu) into 8-week-old ROSA-mTomato mice (The Jackson Laboratory).

### Isolation, flow cytometry, fluorescence-activated cell sorting (FACS) and magnetic activated cell sorting (MACS)

Primary mouse HSCs and MHs were isolated using a two-step collagenase perfusion protocol, as described previously 11, 15. For flow cytometry, cells were incubated with PE/Cy7-conjugated anti-mouse CD24 antibody (Biolegend, 101822) at 4 °C for 30 min, or were fixed with Fixation and Permeabilization Solution (BD, 6292704) at 4 °C for 20 min and then incubated with primary antibodies (CK19, 1:200, rabbit polyclonal, Proteintech, 10712-1-AP; Hnf4α, 1:100, mouse polyclonal, Abcam, ab41898), followed by secondary antibodies. After staining, cells were analyzed on a Beckman MoFlo XDP (Beckman). Suspensions of single-cells were analyzed for the mTom^+^ marker and sorted on a Beckman MoFlo XDP equipped with 405, 488, 561, and 640 nm excitation lasers, as described previously 14. To isolate CD24^+^ progenitor cells, single cell suspensions were prepared from mouse liver using a gentle MACS dissociator (Stemcell), followed by selection with APC-conjugated anti-mouse CD24 (Biolegend, 101814) microbeads in accordance with the manufacturer's instructions (EasyStep Mouse APC Positive Selection Kit, Stemcell).

### Staining and imaging

Paraffin-embedded tissue sections (3 μm) were deparaffinized and rehydrated in a graded series of alcohol concentrations. The following primary antibodies were used for immunohistochemistry (IHC): α-SMA (1:50, rabbit polyclonal, Abcam, ab5694), GFP (1:100, rabbit polyclonal, Abcam, ab183734), and ki67 (1:600, rabbit polyclonal, Servicebio, GB111141). Sections were stained with a Sirius Red/Fast Green (Chondred) and antibody for IHC or H&E using routine protocols.

For immunofluorescence staining, liver tissue was fixed overnight at 4 °C in 4% paraformaldehyde, immersed in a graded series of sucrose solutions, embedded in OCT medium (Tissue Tek) and stored at -80 °C. Frozen sections were then prepared and incubated with the following antibodies: CD24 (1:200, mouse polyclonal, Biolegend, 101801; 1:200, mouse polyclonal, Santa Cruz, sc-19585), Alb (1:200, rabbit polyclonal, Proteintech, 16475-1-AP), CK19 (1:200, rabbit polyclonal, Proteintech, 10712-1-AP), Hnf4α (1:100, mouse polyclonal, Abcam, ab41898), and α-SMA (1:100, mouse polyclonal, Sigma, A5228). Representative images of Sirius Red, H&E, and IHC staining were captured with a Leica Aperio AT Turbo. Images of immunofluorescence staining were captured with a Leica TCS SP8. Sirius staining was quantified by Image J software.

### Human liver biopsies

Human liver biopsies were obtained from patients diagnosed with liver fibrosis and hepatic hemangioma at the Shanghai Eastern Hepatobiliary Surgery Hospital (Supplemental Table S1, S2). Informed consent was obtained from each patient, and the study protocol was approved by the Clinical Research Ethics Committee of Shanghai Eastern Hepatobiliary Surgery Hospital. A diagnosis of liver fibrosis was confirmed by histological examination.

### RNA sequencing and bioinformatics analysis

Total RNA was isolated using an RNeasy mini kit (Qiagen, Germany) and quantified with a NanoDrop ND-2000 spectrophotometer (Thermo Fisher Scientific, Waltham, MA, USA). RNA integrity was determined using an Agilent 2100 system and an RNA 6000 Nano kit (Agilent Technologies, Santa Clara, CA, USA). Paired-end libraries were then constructed with a TruSeq Stranded mRNA LTSample Prep Kit (Illumina, San Diego, CA, USA) in accordance with the manufacturer's instructions. The libraries were then sequenced on an Illumina platform (HiSeq X Ten, Illumina, Shanghai OE Biotech. Co., Ltd.) and 150 base pair (bp) reads were generated.

Raw data (raw reads) were processed using Trimmomatic. Reads containing poly-N sequences and low quality reads were removed so that we could acquire clean reads. Next, the clean reads were mapped to the reference genome using hisat2. The FPKM value of each gene was calculated using cufflinks, and read counts for each gene were obtained by htseq-count. Original data were uploaded to the Gene Expression Omnibus database (gene sets GSE135951 and GSE125095). Gene expression profile analyses included transcriptomic data from two MH gene sets (GSM4101070 and GSM4101071). DEGs were identified using the DESeq (2012) functions estimateSizeFactors and nbinomTest in the R package. KEGG pathway enrichment analysis was carried out for all DEGs using the hypergeometric distribution in R software.

### The determination of liver function parameters

Liver dysfunction was defined according to standard liver biochemistry tests. Serum aspartate aminotransferase (AST) and alanine aminotransferase (ALT) were analyzed using the IFCC method on an Automatic Biochemical Analyzer (Au2700, Olympus, Japan). Total collagen content was then tested by measuring the amount of hydroxyproline in liver tissue using a commercially available hydroxyproline detection kit purchased from NanJingJianCheng Biochemical Institute (Nanjing, China); this was used in accordance with the manufacturer's instructions.

### Quantitation and statistical analysis

All statistical analyses were performed using GraphPad Prism 7 from at least three independent experiments. The two-tailed unpaired t-test was used to compare the statistical significance between two mean values. One-way analysis of variance (ANOVA) was used with Dunnett's correction for the multiple comparisons of multiple values to a single value. All data are presented as mean ± standard deviation and p < 0.05 was considered to be statistically significant.

## Results

### Transplantation of CD24^+^ HepLPCs attenuated liver fibrosis

**Figure 1:**
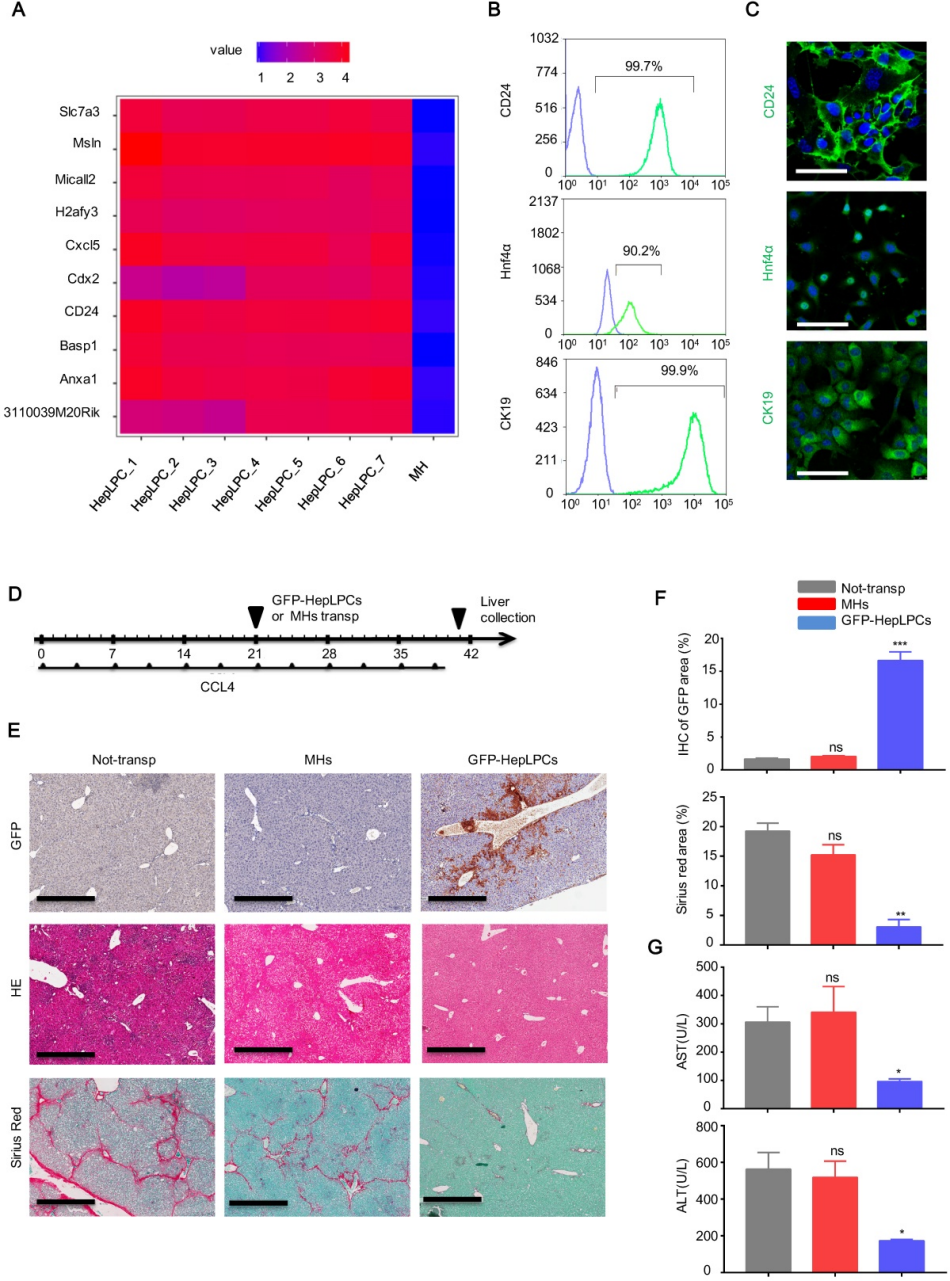
** Transplantation of CD24^+^ HepLPCs effectively attenuated liver fibrosis.** (A) Heat map representation of the top 10 differentially expressed genes in HepLPCs and MHs. (B, C) Flow cytometric and immunofluorescence analysis showing the proportions of CD24-, Hnf4α-, and CK19-positive cells in sample populations. For immunofluorescence, green represents positive-cells and blue represents negative controls. Scale bars, 50 µm. (D) Schematic overview of the experimental setup. Eight-week-old wild-type C57 mice were injected (i.p.) with CCL4 (2 mL/kg, Sigma-Aldrich) dissolved in olive oil at a ratio of 1:4 (2 mL/kg) twice a week for 6 weeks. For cell transplantation, GFP-HepLPCs or MHs (1-2x10^6^, 200 µL of William E medium) were injected intrasplenically after 3 weeks of CCL4 induction (HepLPCs and MHs group); 200 µL of William E medium was used as a control (the 'non-transp' group). After transplantation, all CCl4-injected mice were treated with 2 mL/kg of CCl4, twice a week for 3 weeks, n=5 /group. (E, F) The transplantation of CD24^+^ HepLPCs reduced CCL4-induced liver fibrosis and restored liver function. The transplantation of CD24^+^ HepLPCs or MHs is described in D; Liver fibrosis was confirmed by H&E staining and Sirius red staining; IHC of GFP for CD24 expression, scale bars, 600 µm. Quantification of positive-staining areas was measured by Image J software. (G) Samples of serum were harvested for the analysis of ALT and AST. For F-G, results are shown as mean ± s.d. of three independent experiments. Error bars represent s.d.; *p < 0.05, **p < 0.01, ***p < 0.001, ns represents no significance.

Based on bioinformatics analysis of the gene expression profiles of HepLPCs 11, we found that CD24, an important cell surface marker of progenitor cells, was listed in the top 10 differentially expressed genes of a heat map produced from HepLPCs (Figure 1A). This was verified by flow cytometry and immunofluorescence (Figure 1B-C). In addition, both CK19, a marker of biliary epithelial cells (BECs), and Hnf4α, a key hepatic lineage marker, were expressed in HepLPCs, further suggesting their progenitor properties (Figure 1B-C).

Next, we investigated whether HepLPCs possessed the capability to promote liver recovery from chronic damage. A lentivirus vector carrying GFP was used to generate GFP-positive HepLPCs. After 3 weeks of CCL4 induction, GFP-HepLPCs or MHs (1-2×10^6^) were injected intrasplenically into mice with fibrosis. As a control (non-transp), William E medium was transplanted, using the method described above. All CCl4 injected mice were treated with 2 mL/kg of CCl4, twice a week for 3 weeks after transplantation (Figure 1D). Livers were harvested and analyzed on day 40; GFP-positive cells were still detectable in mice transplanted with CD24^+^-HepLPCs (Figure 1E-F). The transplantation of GFP-HepLPCs significantly reduced the size of the fibrotic areas and reduced the serum levels of ALT and AST in CCL4-treated mice (Figure 1E-G). Together, these results suggest a therapeutic effect of small molecule-induced CD24^+^ HepLPCs in the treatment of chronic liver fibrosis.

### Endogenously induced CD24^+^ cells mitigated liver fibrosis

**Figure 2:**
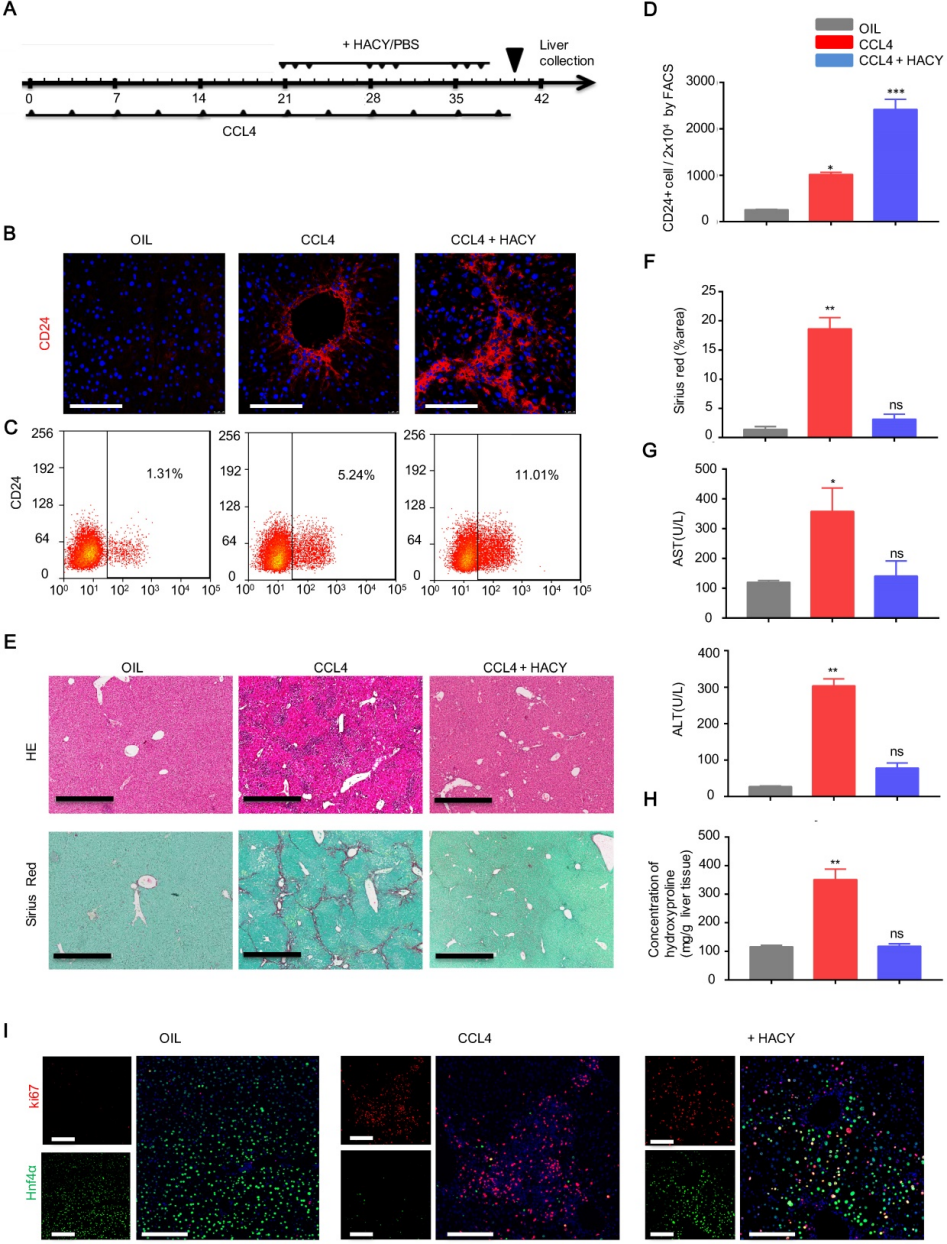
** HACY treatment increased the proliferation of CD24^+^ cells to attenuate liver fibrosis upon CCL4 treatment.** (A) Schematic outline showing chronic CCL4 damage. Eight-week-old WT mice were injected with CCL4 (2 mL/kg, dissolved in olive oil at a ratio of 1:4) and HACY (HGF, A-83-01, CHIR99021 and Y-27632, 1 µg/molecules/mice/time, dissolved in 100 µL PBS) or PBS (100 µL, CCL4 group); olive oil was used as a control (2 mL/kg, OIL group), n=5 /group. (B) CD24 expression, as determined by immunofluorescent assays with a specific antibody on day 40, scale bars, 200 µm. (C, D) CD24 expression, as determined by FACS assay. (E, F) Livers were harvested and stained using H&E and Sirius Red to allow the analysis of fibrosis, scale bars, 600 µm. (F) The quantification of areas showing positive-staining was carried out by Image J software. (G) Serum was harvested for the analysis of ALT and AST. (H) Quantification of hepatic hydroxyproline content. (I) The co-expression of ki67 and Hnf4α, as demonstrated by immunofluorescence analyses, scale bars, 200 µm. For D, F, G, H, the results are shown as the mean ± s.d. of three independent experiments. Error bars represent s.d.; *p < 0.05, **p < 0.01, *** p < 0.001, ns represents no significance.

Next, we explored how to induce more endogenous CD24^+^ liver progenitor cells* in vivo*. Our previous studies demonstrated that TEM was composed of a cocktail of growth factors and small molecules containing HGF, EGF, CHIR99021, LPA, S1P, A83-01 and Y27632. Of these, HACY (HGF, CHIR99021, A83-01 and Y27632) was found to be crucial for hepatocyte-to-LPC conversion 12, 14. We therefore investigated whether the combination of HACY could efficiently induce CD24^+^ cells *in vivo*. For this purpose, HACY at a dose of 1 μg or PBS were injected (i.p.) into CCL4-treated mice, three times a week for 3 weeks; olive oil was used as a control (Figure 2A). Immunofluorescence and flow cytometry analysis with an anti-CD24 antibody demonstrated that the administration of HACY significantly increased the number of CD24^+^ cells in CCL4-treated C57 mice compared with those injected with PBS (Figure 2B-D).

Furthermore, we observed that HACY treatment efficiently reduced the size of the fibrotic area when compared with control mice (Figure 2E-F). The serum levels of ALT and AST, and the concentration of hydroxyproline, a marker of fibrosis, in the HACY-treated mice were also significantly decreased compared with their levels in control mice (Figure 2G-H); these findings were in line with the results obtained from our HepLPCs transplantation experiments. To ascertain the potency of HACY for host hepatocytes *in vivo*, ki67 staining was used to examine the proliferative ability of hepatocytes. Notably, the number of regenerated hepatocytes that co-expressed ki67 and Hnf4α was significantly higher in the HACY group than in the CCL4 group (Figure 2I). Taken together, these results provide evidence that the *in vivo* administration of HACY can alleviate liver injury and promote regeneration.

### HACY-induced endogenous CD24^+^ cells are transcriptionally and functionally distinct from CCL4-induced cells

**Figure 3:**
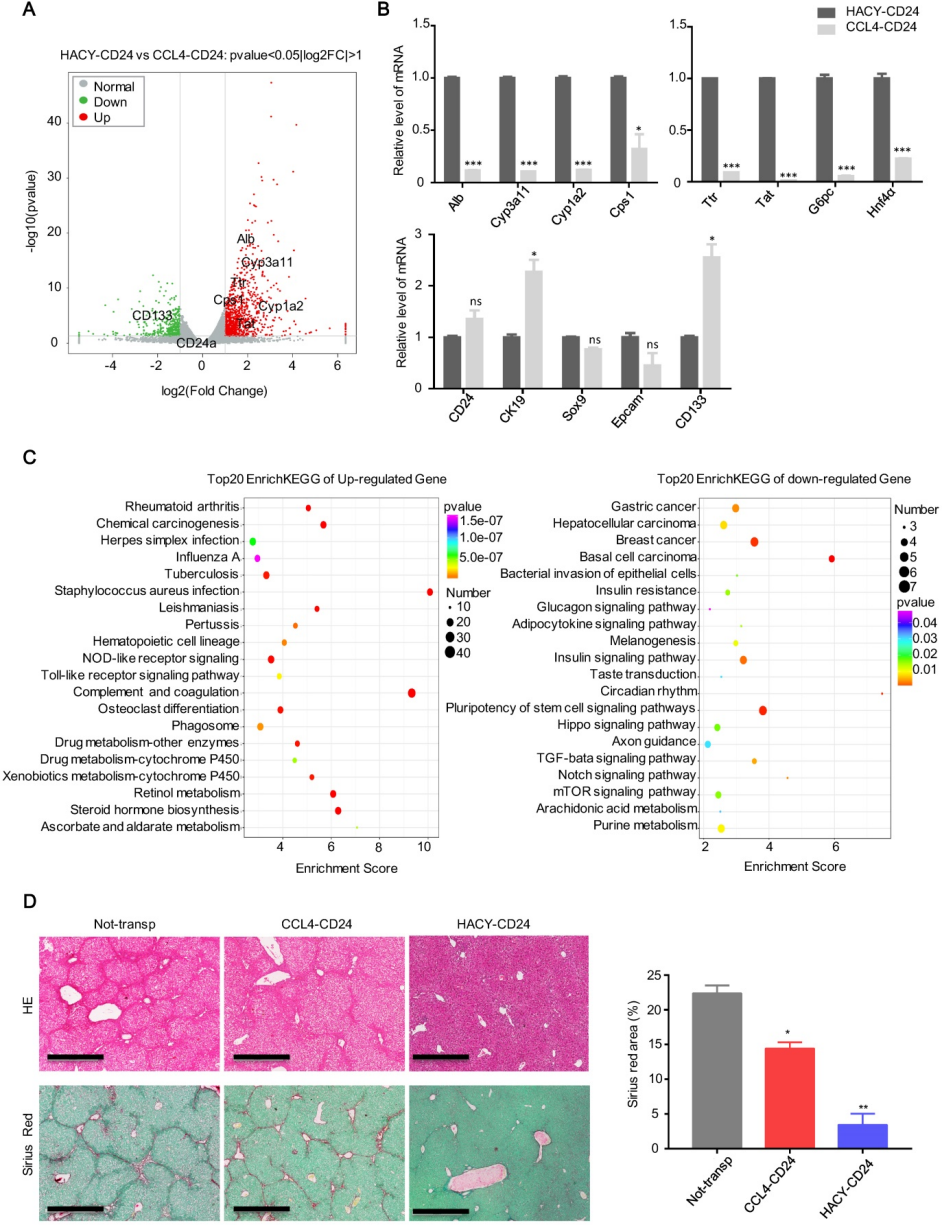
** HACY-induced endogenous CD24^+^ cells are transcriptionally and functionally distinct from CCL4-induced cells.** (A) Correlation scatter plot showing the changes of related genes in CD24^+^ progenitor cells, including HACY-CD24^+^ and CCL4-CD24^+^ (isolated from the model of liver fibrosis with or without HACY treatment). Each element represents log2 (normalized expression), as scaled by the corresponding color legends. n = 2 independent experiments. (B) qRT-PCR analyses of hepatic marker genes in HACY-CD24^+^ and CCL4-CD24^+^ from models of liver fibrosis with or without HACY treatment. (C) KEGG pathway analysis comparing the top 20 between HACY-CD24 cells and CCL4-CD24 cells, as indicated by the color legend. (D) The transplantation of HACY-CD24 cells and CCL4-CD24 cells is described in Figure 2A; controls were treated with William E medium; Liver fibrosis was confirmed by H&E staining and Sirius red staining, Scale bars, 600 µm; The quantification of areas showing positive-staining with Sirius red was carried out by Image J software. For B, D, the results are shown as mean ± s.d. of three independent experiments. Error bars represent s.d.; *p < 0.05, **p < 0.01, *** p < 0.001, ns represents no significance.

Global expression analysis of RNA sequencing data was used to compare the expression levels of MACS-sorted CD24+ cells from CCL4 with or without HACY treatment (which we referred to as HACY-CD24^+^ cells and CCL4-CD24^+^ cells). When compared to CCL4-induced CD24^+^ cells, 848 genes were found to be significantly up-regulated in HACY-CD24^+^ cells while 369 genes were down-regulated (Figure S1A-B). Notably, gene sets related to hepatic function were up-regulated in HACY-CD24^+^ cells when compared with CCL4-CD24^+^ cells (Figure 3A). Consistent with our volcano plots, quantitative gene expression analyses indicated that HACY-CD24^+^ cells not only retained the expression of hepatocyte-lineage marker genes, including Alb, Cyp3a11, Cps1, and Hnf4α, but also exhibited the expression of hepatic progenitor cell markers such as Sox9, CK19, and CD24 (Figure 3B). Moreover, HACY-CD24^+^ cells showed enrichment in the expression of genes involved in the metabolism of xenobiotics by cytochrome P450 and drug metabolism (Figure 3C). Collectively, transcription profiles indicated that there was a distinction between CD24^+^ progenitor-like cells induced by CCL4 and CCL4 plus HACY. HACY-induced CD24^+^ cells retained basal levels of transcription for genes encoding mature hepatocyte functions.

Further transplantation studies were performed to confirm the *in vivo* potency of HACY- and CCL4-induced endogenous CD24^+^ liver progenitor cells. We intrasplenically transplanted 1×10^6^ of each type of cell into the CCL4-induced liver fibrosis model shown previously in Figure 1D. Groups of HACY-CD24^+^ cells were detected with reduced areas of fibrosis when compared to mice transplanted with CCL4-CD24^+^ cells and control groups on day 40 (Figure 3D). Therefore, these results provided evidence that endogenous CD24^+^ cells induced by HACY exhibited a higher therapeutic potency for the treatment of chronic liver fibrosis, possibly due to better maintenance of hepatic function 16.

### HACY-induced endogenous CD24^+^ cells exhibited a comparable gene expression profile with HepLPCs

**Figure 4:**
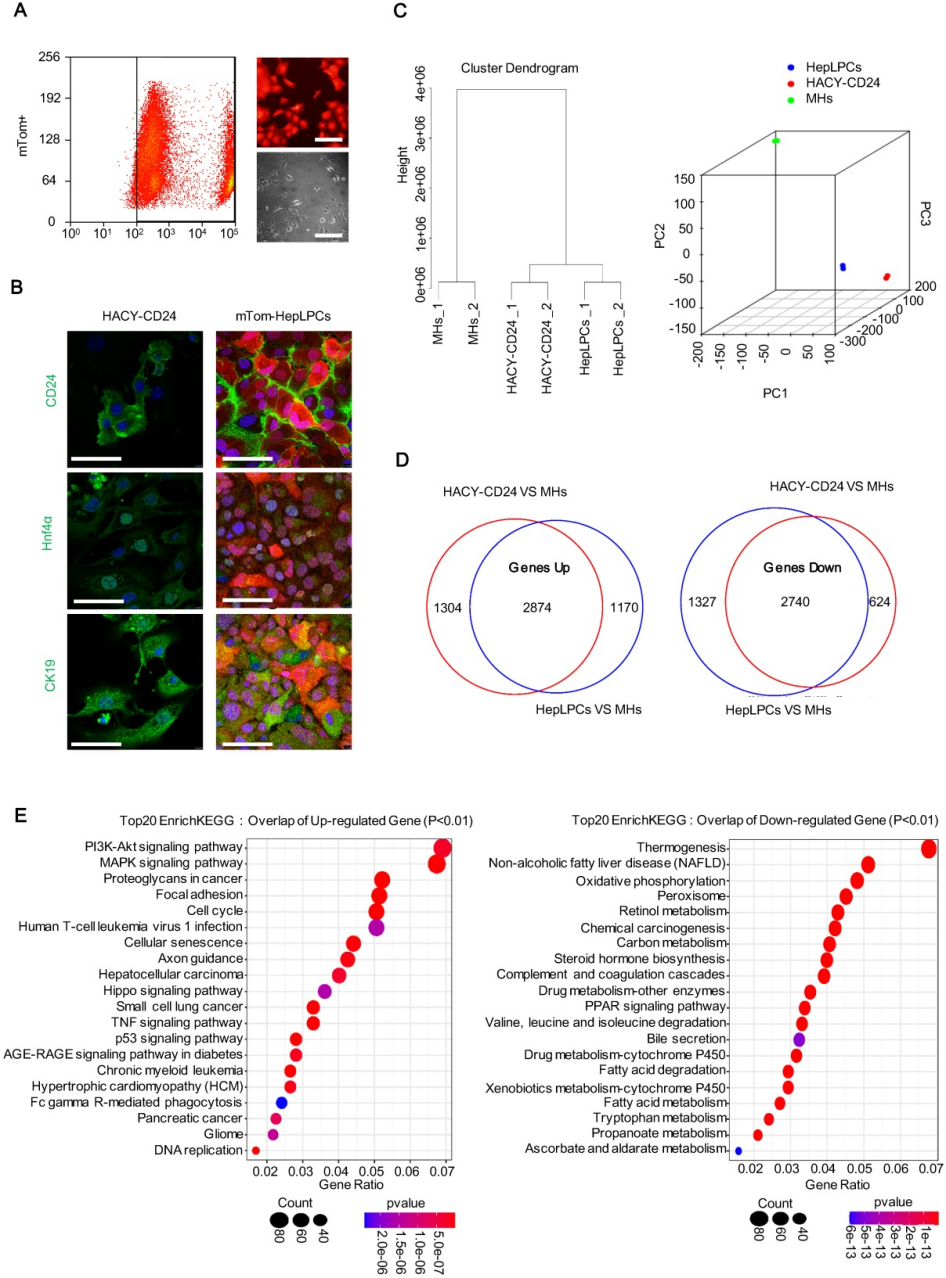
** HACY-induced endogenous CD24^+^ cells showed comparable gene expression profiles with *in vitro* cultured HepLPCs.** (A) Purified fluorescently marked hepatocytes underwent successful expansion as progenitor like cells in TEM *in vitro*. td-Tomato mice were injected with AAV-TBG-Cre virus; subsequently, tom-fluorescently marked hepatocytes (mTom^+^) were isolated by FASC and then cultured in TEM *in vitro*. Light and fluorescent microscopy images of mTom^+^ HepLPCs. Scale bar, 100 µm. (B) A comparison of immunofluorescence analyses of CD24, Hnf4α, and CK19, between CD24^+^ progenitor cells (isolated from liver fibrosis models with HACY) and tom-fluorescently marked HepLPCs (isolated from td-Tomato mice with AAV-TBG-Cre virus and then cultured in TEM *in vitro*), Scale bar, 50 µm. (C) Unsupervised non-hierarchical clustering of samples and principal-component analysis was performed to compare global gene expression profiles in CD24^+^ progenitor cells, mTom-HepLPCs, and mature hepatocytes (MHs). Each element represents the log2 (normalized expression), as scaled by the corresponding color legends. n = 2 independent experiments. (D, E) For D, Venn diagram of results from up- and down-regulated gene expression profiles of CD24^+^ progenitor cells and mTom-HepLPCs compared with MHs respectively. Red regions represented the numbers of genes in HACY-CD24^+^ cells compared with MHs, while blue regions represented the numbers of genes in mTom-HepLPCs compared with MHs. The intersection between the red and blue regions represented genes where the expression levels increased or decreased simultaneously. For E, Enrichment analysis of the top 20 KEGG pathway in the intersection of the genes lying between the red and blue regions.

To compare the transcriptional profile of the cultured HepLPCs *in vitro* and the HACY-induced CD24^+^ cells *in vivo*, mature hepatocytes were first genetically labelled by administering td-Tomato mice with adeno-associated viruses expressing Cre recombinase under the regulation of the thyroxine-binding globulin promoter (AAV-Cre-TBG). This promoter specifically and efficiently converted mTom-negative hepatocytes (mTom^-^) into mTom-positive (mTom^+^) hepatocytes. Following sorting by FACS, purified mTom^+^ hepatocytes were able to proliferate in TEM with the typical features of liver progenitor-like cells; we referred to these cells as mTom^+^ HepLPCs (Figure 4A). HACY-induced endogenous CD24^+^ cells were isolated by MACS from CCL4-treated mouse liver. HACY-CD24^+^ cells and mTom+ HepLPCs showed similar expression levels of CD24, Hnf4α, and CK19, as determined by immunofluorescence analyses (Figure 4B).

Further characterization was supported by global expression analysis of RNA sequencing data to compare the gene expression levels of mTom^+^ HepLPCs and HACY-CD24^+^ cells. MHs were included as controls. Unsupervised non-hierarchical clustering of samples, along with principal-component analysis, revealed that HACY-CD24^+^ cells clustered closely with mTom^+^ HepLPCs (Figure 4C). The overlap of a Venn diagram showed that a set of 2874 genes in both HACY-CD24^+^ cells and mTom-HepLPCs showed increased expression levels when compared with MHs, while 2740 overlapping genes showed reduced levels of expression, accounting for over 65% of all the differentially expressed genes (Figure 4D). Moreover, enrichment analysis of the top 20 KEGG pathways showed that the up-regulated genes were enriched in pathways related to cell proliferation, growth, and development, including the PI3K-Akt and Hippo signaling pathways, thus demonstrating that these cells retained the characteristics of liver progenitor cells (Figure 4E). Taken together, the endogenously induced HACY-CD24^+^ cells exhibited similar expression profiles with *in vitro* cultured HepLPCs.

### HACY treatment suppressed the activation of hepatic stellate cells during liver fibrosis

**Figure 5:**
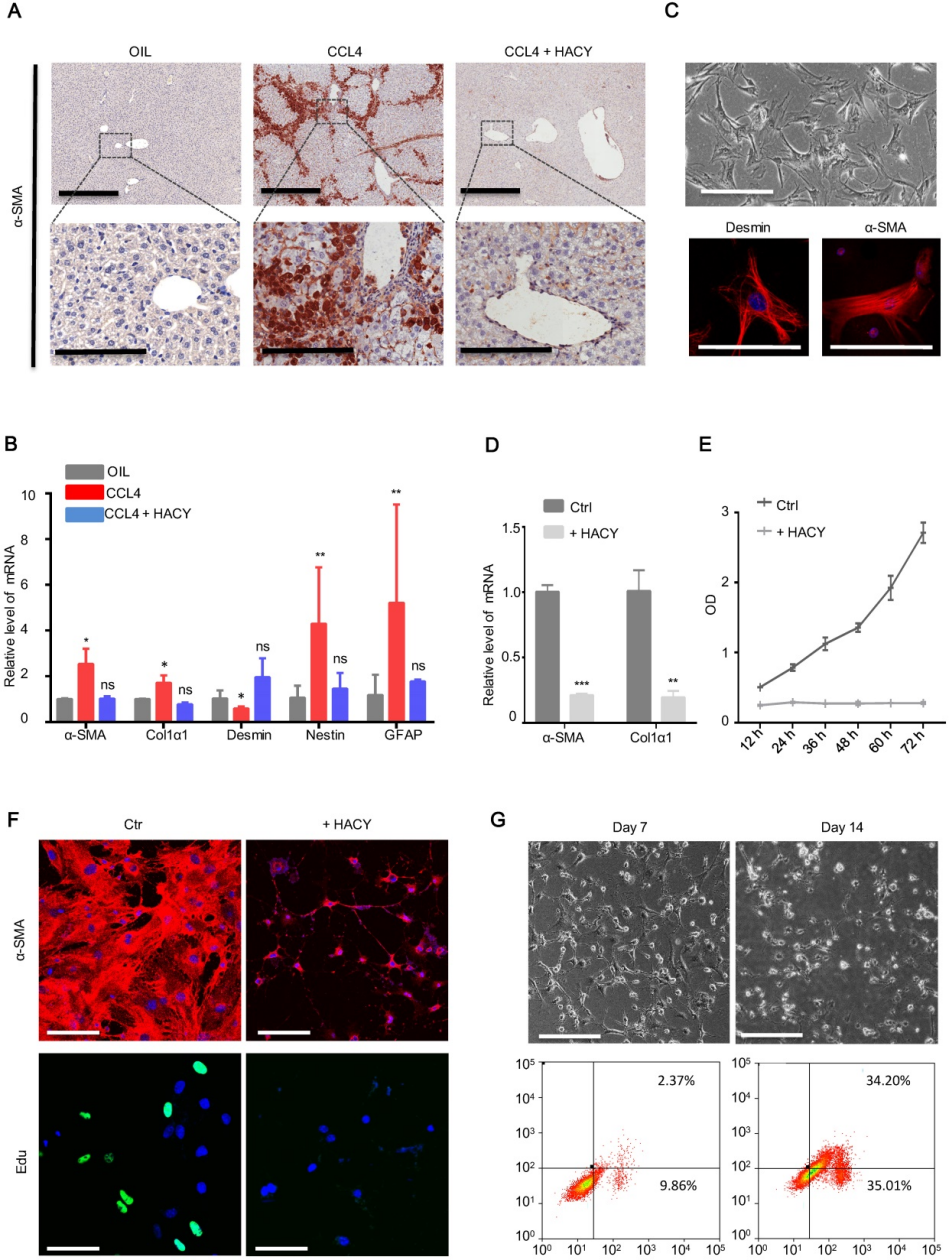
** HACY conferred the suppression of hepatic stellate cells during liver fibrosis.** (A) IHC of liver fibrosis models with or without HACY treatment at 40 days for α-SMA expression, Scale bars, 600 µm (top); 200 µm (down). (B) qRT-PCR analysis of hepatic stellate cells genes for α-SMA, Col1α1, Desmin, Nestin, and GFAP, in the liver tissues. (C) Morphology of mHSCs in bright field images (top), scale bars, 100 µm. The expression of Desmin and α-SMA were analyzed by immunofluorescent assay (bottom); results represent three separate experiments, scale bars, 50 µm. (D) α-SMA and Col1α1 expression in primary mHSCs with or without HACY on day 3, as determined by qRT-PCR analysis. (E) Growth curves of mHSCs with or without HACY at different time points, as determined by CCK8 assays. (F) Expression of α-SMA by immunofluorescence and Edu incorporation in primary mHSCs with or without HACY on day 7. Scale bars, 50 µm. (G) Morphology of mHSCs on day 7 and 14 in bright field images (top), scale bars, 100 µm. The apoptotic rates of both groups were measured by flow cytometry. For B, D, E, results are shown as mean ± s.d. of three independent experiments. Error bars represent s.d.; *p < 0.05, **p < 0.01, *** p < 0.001, ns represents no significance.

It is generally accepted that activated HSCs are the principal cellular factors that promote fibrosis in response to the accumulation of inflammatory signals derived from damaged parenchymal cells 15. We tested whether HACY treatment could exert effect on the activation of HSCs at a wound site. Mice receiving HACY treatment showed a significant decline in the mRNA and protein levels of α-SMA (Figure 5A, S2A-B), a marker that is characteristic of activated HSCs 17, thus suggesting that HACY had a direct effect on the state of HSCs *in vivo*. q-PCR analyses confirmed that HACY intervention reduced the levels of HSC activation markers 18, including α-SMA, Col1a1, Fibronectin, Desmin, and GFAP (Figure 5B).

To further evaluate the functional role of HACY in HSC quiescence/inactivation, primary mice HSCs were isolated and cultured *in vitro* (Figure 5C). In the presence of Y-27632 and A-83-01, the proliferative ability of HSCs was impaired but in a far less significant manner than the influence of HACY (Figure S2C-D); this correlated with a reduction of α-SMA and Col1α1 expression relative to the control group on day 3(Figure 5D-E). Further evidence to support the inactivation of HSCs by HACY on day 7 was acquired by immunofluorescence and Edu assays (Figure 5F). When *in vitro* culture time was prolonged to day 14, there was an obvious increase in the rate of apoptosis (Figure 5G). Collectively, these data indicated that the clearance of activated HSC took place after HACY treatment.

### HACY triggered the expansion of CD24^+^ cells in hepatic spheroids from human fibrotic tissues

**Figure 6:**
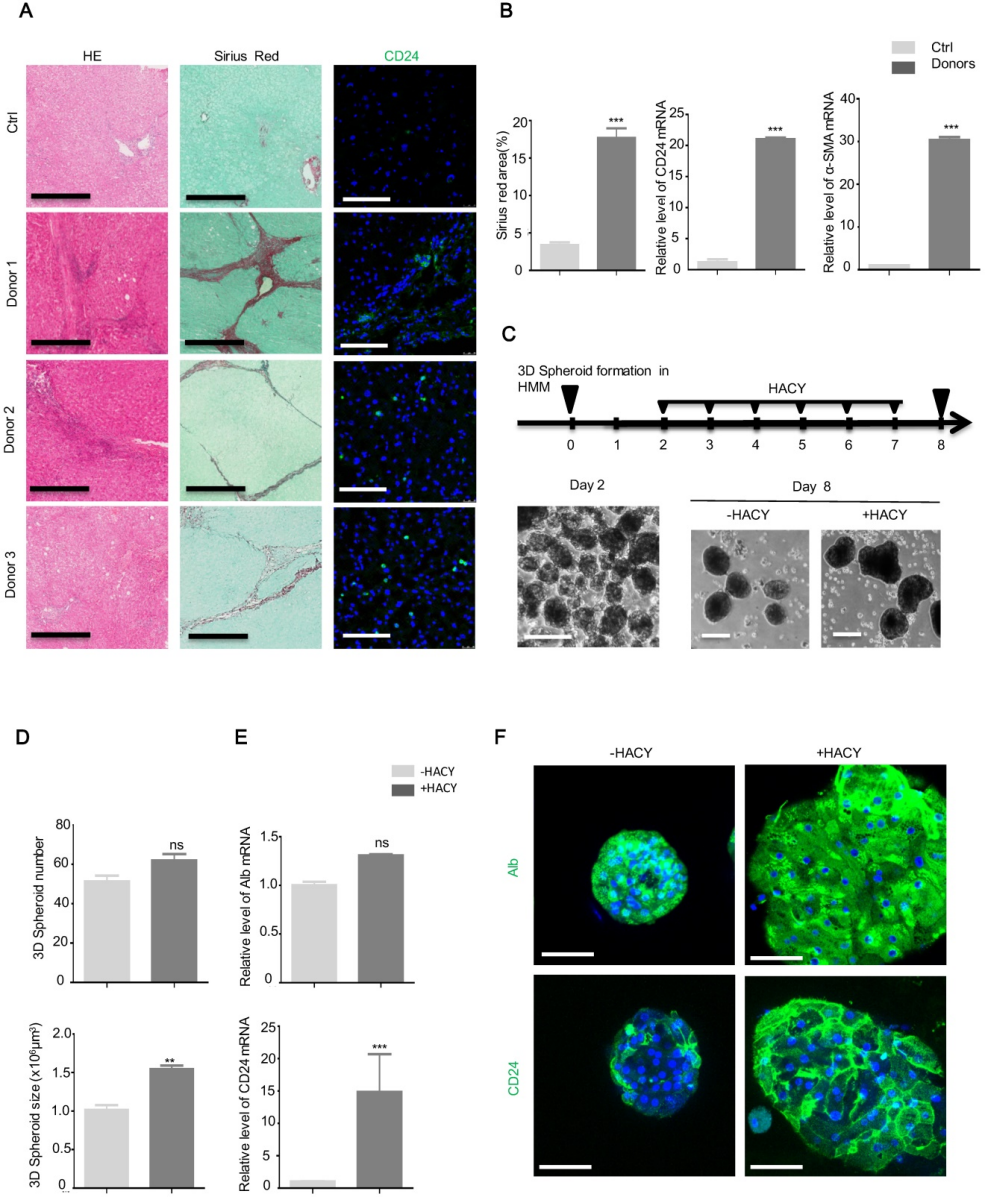
** CD24^+^ progenitor cells were detectable in human liver fibrosis tissues.** (A) CD24, Sirius Red, and H&E, staining in human liver samples. Scale bars, 600 µm for H&E staining and Sirius red staining; 200 µm for immunofluorescence for CD24. (B) Quantification of areas showing positive-staining for Sirius red was carried out using Image J software. mRNA levels of CD24 and α-SMA were determined by qRT-PCR analysis in human liver samples. (C) A schematic overview of the experimental setup (top) and representative images (bottom). Scale bars, 100 µm. (D) Hepatic spheroid numbers and size, as determined on day 8. (E) qRT-PCR analysis of Alb and CD24 in representative hepatic spheroids. (F) Immunofluorescence analyses demonstrating the expression of Alb and CD24. Scale bars, 50 µm. For B, D, E, the results are shown as mean ± s.d. of three independent experiments. Error bars represent s.d.; **p < 0.01, *** p < 0.001, ns represents no significance.

Finally, we obtained samples from seven patients with clinically diagnosed liver fibrosis (Supplementary Table 1) and five normal liver samples with hepatic hemangioma as controls (Supplementary Table 2). Using an antibody against CD24 for immunofluorescence, CD24-positive cells were observed in the samples of liver fibrosis but were undetectable in samples of normal liver (Figure 6A). The enhanced expression of CD24 and α-SMA were confirmed by qPCR assays (Figure 6B).

Since the three-dimensional (3D) culture of hepatocytes is essential to reconstruct functional hepatic tissues *in vitro* 19, we isolated primary MHs from three biopsies of fresh liver fibrosis and cultured the biopsied tissue on a low-attachment cell plate with gentle shaking to form 3D spheroids. Next, we investigated whether CD24^+^ progenitor cells could be induced by HACY in these spheroids. After 2 days of HMM culture, the isolated hepatocytes successfully generated 3D cell spheroids; these were further cultured in the presence or absence of HACY (Figure 6C). It is of note that HACY-treated spheroids exhibited proliferative advantages compared to non-treated spheroids after 7 days of culture in terms of number and size (Figure 6D). In accordance, HACY induced higher expression levels of CD24 mRNA (Figure 6E) and gave rise to a greater number of human CD24^+^ progenitor cells in the spheroids than control groups (Figure 6F). Collectively, these results showed that CD24^+^ progenitor cells existed in human liver fibrosis tissues. Treatment with HACY further induced the generation of CD24^+^ progenitor cells in 3D hepatic spheroids.

## Discussion

Under chronic pathological conditions, a reversible transition from hepatocytes to liver progenitor cells could occur in an attempt to alleviate various chronic hepatic injuries; these progenitor cells have the capacity to differentiate into hepatocytes *in vivo* 20. Therefore, the induction of endogenous adult stem cells by administering soluble molecules provides an advantageous approach for aiding in the replenishment of parenchymal cells and the revitalization of regeneration. A recent study showed that the application of Rspo1, a molecule that promotes lgr5^+^ liver stem cells *in vitro*, along with VPA and EPZ6438, was indispensable for the culture of hyperplastic intestinal organoids and could significantly inhibit liver fibrosis and promote regenerative responses in the intestinal epithelium after irradiation* in vivo* 9, 21. In line with these findings, we observed that the administration of HACY, which mimics the regeneration milieu, facilitated the induction of liver progenitor cells in mice with liver dysfunction, as well as in hepatic spheroids isolated from patients diagnosed with liver fibrosis.

However, CD24^+^ progenitor cells could be induced both by CCL4 with or without HACY, contributing to different outcomes and prognoses of fibrosis. CCL4-CD24^+^ cells showed little therapeutic benefit, consistent with the observation that a correlation has been observed in the degree of LPC activation and the severity of liver disease in chronic human liver diseases 22. In contrast, our findings suggest that both HACY-induced CD24^+^ cells and *in vitro*-cultured HepLPCs could contribute to regeneration during persistent injury. Based on RNA sequencing data and bioinformatics analysis, we demonstrated that HACY-induced CD24^+^ cells retained basal levels of transcription for genes encoding mature hepatocyte functions, including Alb expression, fatty acid metabolism, complement and coagulation cascades, drug metabolism, and cytochrome P450. Therefore, it is conceivable that the induction of hepatic parenchymal metaplasia by soluble molecules might enhance the fitness of hepatocytes to fibrotic stimuli without genetic modification. In agreement with endogenous HACY-induced CD24^+^ cells displaying close clustering with HepLPCs in our gene expression profiling, our ongoing experiments demonstrated that the expression of α-SMA in HCSs was diminished in the presence of HepLPCs, both *in vivo* and *in vitro*, thus suggesting that HepLPCs had a significant effect on the conversion of myofibroblasts to quiescent HSCs.

Liver fibrosis arises from the wound-healing response to repeated injury. If the hepatic injury persists, the regeneration of parenchymal cells eventually fails, leading to persistent hepatocyte apoptosis and the activation of HSCs 23. Since the elucidation of fibrosis requires multi-targeted efforts, rational treatment approaches for liver fibrosis may include drugs that target hepatocyte apoptosis and stellate cell activation, simultaneously 10. These anti-fibrotic drug cocktails are likely to target a variety of orthogonal mechanisms, including a range of receptors, signaling pathways, and cell types that have been shown to function as core drivers of fibrosis in multiple disease states 24. We have provided evidence that the administration of a cocktail of soluble molecules could induce the proliferation of CD24^+^ progenitor cells and the clearance of myofibroblasts *in vivo*. This facilitated the replenishment of parenchymal cells and accelerated the restoration of fibrotic tissue. Notably, native hepatocytes exhibited a marked increase in Ki67^+^ expression in the liver following HACY administration, thus showing a potential benefit by stimulating hepatic regeneration in the host. Other studies have shown that *in vivo* EGF treatment can reestablish the balance between growing follicles and dormant follicles following an acute change of ovarian microenvironment 25. Similarly, the internal administration of HACY may reestablish a balance favoring liver regeneration, thus providing a clinically effective approach for the treatment of chronic liver disease.

Compared to the transgenic approach which converted pro-fibrogenic myofibroblasts into hepatocyte-like cells through the AAV vector to prevent fibrosis and improve the outcome of chronic liver disease 26, 27, a chemical approach may be more promising and more likely to progress in the clinic 28. This is because small-molecule compounds are cell permeable, reversible, and easily manufactured. They can also be fine-tuned in terms of concentration, duration, structure, and combination 29. However, whether these anti-fibrotic drug cocktails have side effects remains unknown. Further studies and longer observation periods are now necessary if we are to clarify the specific changes and long term toxicology in general reactivity.

## Conclusion

Here, we provide evidence that HACY treatment can induce the expansion of endogenous CD24+ progenitor cells and the clearance of activated HSCs. Our data shed significant light on the dynamic processes that govern regeneration and fibrosis to reinstall homeostasis in a liver suffering from chronic hepatic injury. These multifaceted approaches should pave the way towards the future delivery of effective anti-fibrotic therapies *via* the internal administration of soluble molecules.

## Supplementary Material

Supplementary figures and tables.

## Abbreviations

CD24: cluster of differentiation 24
Cps1: carbamoyl-phosphate synthase 1
Cyp3a11: cytochrome P450 family 3 subfamily A member 11
Cyp1a2: cytochrome P450 family 1 subfamily A member 2
Ttr: transthyretin
Tat: tyrosine aminotransferase
G6pc: glucose-6-phosphatase, catalytic
CD133: cluster of differentiation 133
Hnf4α: hepatocyte nuclear factor 4-alpha
CK19: Cytokeratin 19
Alb: albumin
α-SMA: alpha smooth muscle Actin
COL1α1: collagen type I alpha 1 chain
MHs: Mature hepatocyte
HepLPCs: hepatocyte-derived liver progenitor-like cells
LPCs: liver progenitor-like cell
mTom: mTomato
GFP: Green fluorescent protein
HE: hematoxylin eosin
CCL4: carbon tetrachloride
TEM: Transition and Expansion Medium
TBG: thyroxine-binding globulin
AST: Aspartate transaminase
ALT: Alanine aminotransferase
FACS: fluorescence activated cell sorting
MACS: magnetic bead cell sorting
DAPI: 4',6-diamidino-2-phenylindole
