# Supplementary material for: The combined induction of liver progenitor cells and the suppression of stellate cells by small molecules reverts chronic hepatic dysfunction
Source: Theranostics. 2021 Mar 14;11(11):5539–52. doi: 10.7150/thno.54457 (PMC8039967; doi:10.7150/thno.54457)
Supplement: Supplementary file 1 — Supplementary figures and tables. [file thnov11p5539s1.pdf]

SUPPLEMENTARY MATERIALS

Wei-Jian Huang[1,2,*], Xu Zhou[1,*],Gong-Bo Fu[1,3,*], Min Ding[4,*], Hong-Ping Wu[1,*], Min Zeng[1], Hong-Dan Zhang[5], Ling-Yan Xu[6], Yi Gao[2,#], Hong-Yang Wang[1,#], and He-Xin Yan[1,2,5,7#]

Author affiliations

1. International Cooperation Laboratory on Signal Transduction, Eastern Hepatobiliary Surgery Hospital, Second Military Medical University, Shanghai, China;
2. Department of Hepatobiliary Surgery II, Guangdong Provincial Research Center for Artificial Organ and Tissue Engineering, Zhujiang Hospital, Southern Medical University ;
3. Department of Medical Oncology, Jinling Hospital, First School of Clinical Medicine, Southern Medical University
4. Department of Interventional Oncology, Renji Hospital, Jiaotong University School of Medicine, Shanghai, China
5. Shanghai Celliver Biotechnology Co. Ltd., Shanghai, China
6. Department of Pharmacy, Shanghai Jiao Tong University Affiliated Sixth People's Hospital, East Campus, Shanghai, China;
7. Shanghai Cancer Institute, Renji Hospital, Shanghai Jiaotong University School of Medicine, Shanghai, China.

* These authors contributed equally to this work.

Supplementary methods

Western blot

The protein extracts were submitted to western blotting standard protocol. Briefly, livers tissues were dissected with 300μl of ice cold lysis buffer rapidly in tube and homogenized with an electric homogenizer, then centrifuged for 20 min at 12,000 rpm at 4°C in a micro centrifuge. Protein concentrations were measured using a Pierce™ BCA Protein Assay Kit (Both from Thermo scientific). Then proteins were subjected to electrophoresis on 8–10% Bis-Tris protein gels and transferred to nitrocellulose membranes (GE healthcare), which were incubated with the primary antibodies, followed by a fluorescently conjugated secondary antibody. The fluorescence density on nitrocellulose membranes was measured on Odyssey CLx Western Blot Detection System (LI-COR Biosciences).

Quantitative real-time PCR (qPCR)

Total RNA of cells was extracted using TRIZOL reagent (Invitrogen) according to the manufacturer's protocols. Real-time PCR analyses were performed using a LightCycler® 96 Real-Time PCR System (Roche) and SYBR Green PCR kit (Roche). Gene transcription was evaluated using the ΔΔCt method normalized to the housekeeping gene actin beta (ACTB). Primer sequences are respectively listed in Table S3.

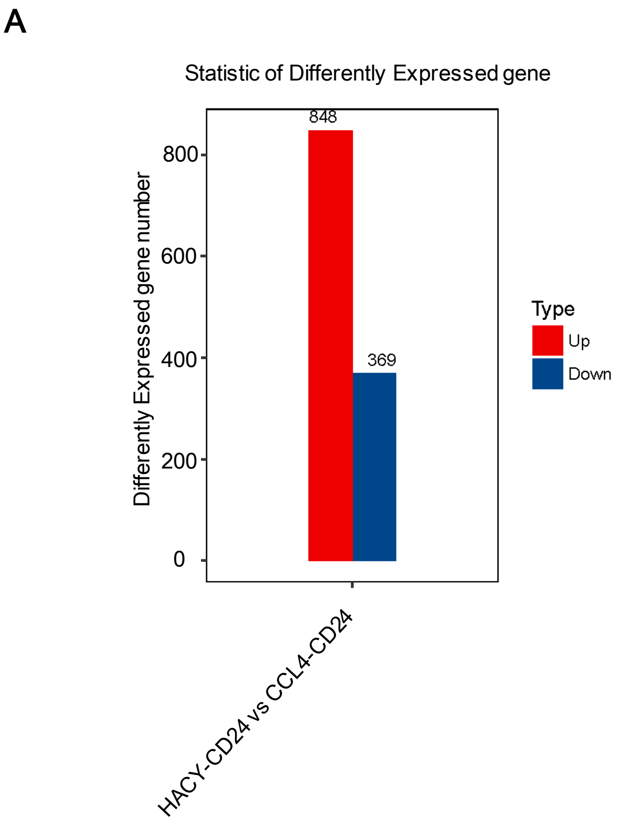

A
Statistic of Differently Expressed gene
848
369
800
600
400
200
0
Differently Expressed gene number
Type
Up
Down
HACY-CD24 vs CCL4-CD24

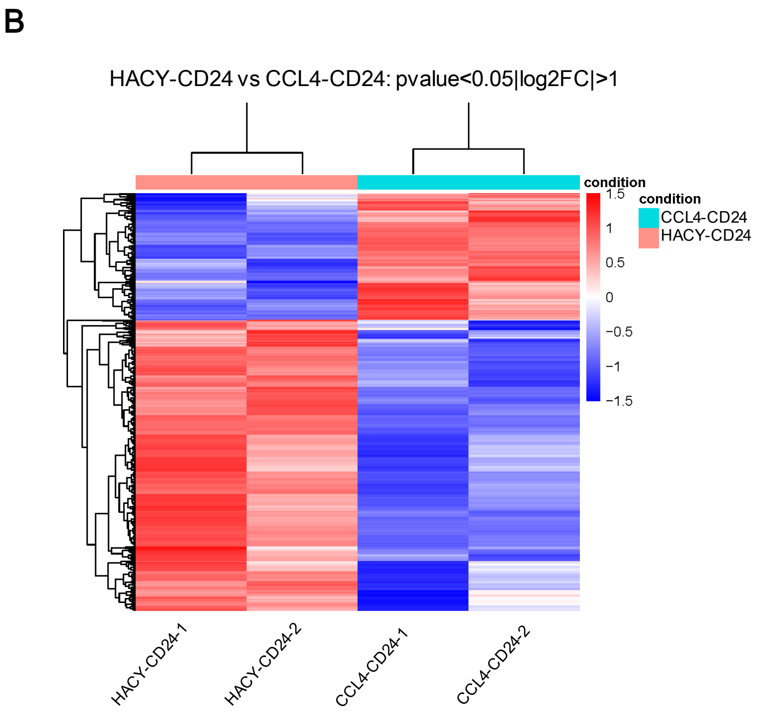

B
HACY-CD24 vs CCL4-CD24: pvalue<0.05|log2FC|>1
condition
1.5
1
0.5
0
−0.5
−1
−1.5
condition
CCL4-CD24
HACY-CD24
HACY-CD24-1
HACY-CD24-2
CCL4-CD24-1
CCL4-CD24-2

Supplementary Figure 1

(A, B) mRNA sequencing was performed to compare global gene expression profiles. Figure depicts a comparison of up- and down-regulated genes ($pv<0.05$ and $|log2FC|>1$) between HACY-CD24 cells and CCL4-CD24 cells. Heat maps for the expression of genes related to hepatic function in CD24+ progenitor cells.

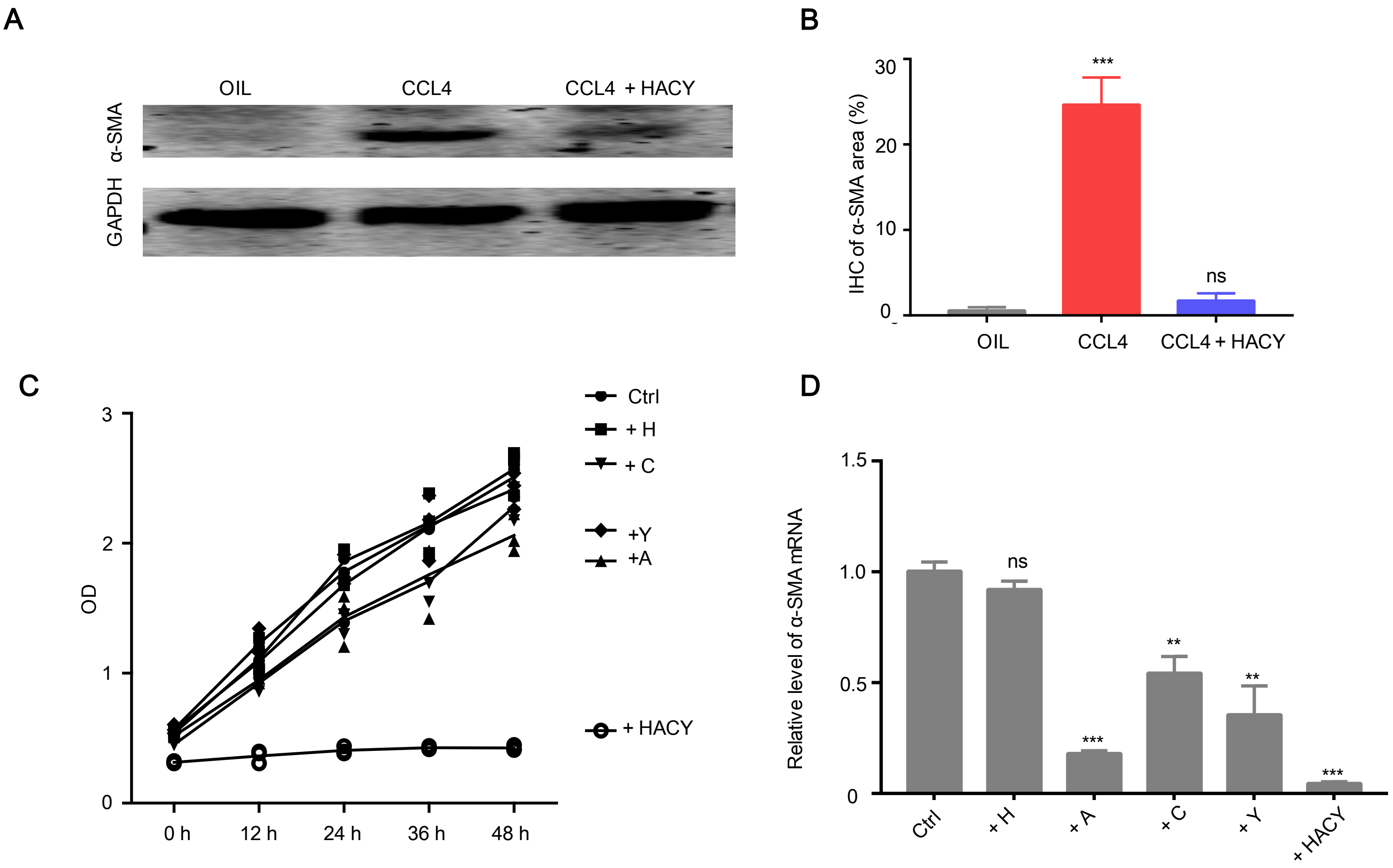

A
OIL
CCL4
CCL4 + HACY
α-SMA
GAPDH
B
30
20
10
0
IHC of α-SMA area (%)
***
ns
OIL
CCL4
CCL4 + HACY
C
3
2
1
0
OD
0 h
12 h
24 h
36 h
48 h
Ctrl
+ H
+ C
+Y
+A
+ HACY
D
1.5
1.0
0.5
0
Relative level of α-SMA mRNA
ns
***
**
**
***
Ctrl
+ H
+ A
+ C
+ Y
+ HACY

Supplementary Figure 2

(A) The protein levels of α-SMA from models of liver fibrosis with or without HACY treatment at 40 days were detected by western blotting. GAPDH was used as an internal control.

(B) Quantification of the α-SMA levels shown in A, as determined by Image J software

(C) Growth curves of mHSCs with HGF, Y-27632, A-83-01, CHIR99021, or HACY, at different time points, as determined by CCK-8 assays. Ctrl represents a blank control.

(D) α-SMA expression in primary mHSCs with HGF, Y-27632, A-83-01, CHIR99021, or HACY on day 3 was determined by qRT-PCR analysis.

For B, D, the results are shown as mean ± s.d. of three independent experiments, $^{**}p < 0.01$, $^{***}p < 0.001$, ns represents no significance

Supplementary table1: Age and gender of the 7 patients with clinically diagnosed liver fibrosis.

Patients Number	Age	Gender
1	78	male
2	51	male
3	75	male
4	56	male
5	50	male
6	57	male
7	67	male

Supplementary table 2: Age and gender of the 5 patients with clinically diagnosed hepatic hemangioma.

Patients Number	Age	Gender
1	49	male
2	50	male
3	63	male
4	61	male
5	32	female

Supplementary table 3: Primer list.

Gene	Forward sequence 5’->3’	Reverse sequence 5’->3’
CD24	GTTGCACCGTTTCCCGGTAA	CCCCTCTGGTGGTAGCGTTA
CK19	GTTCAGTACGCATTGGGTCAG	GAGGACGAGGTCACGAAGC
Hnf4α	ATGCGACTCTCTAAAACCCTTG	ACCTTCAGATGGGGACGTGT
G6pc	CGACTCGCTATCTCCAAGTGA	GGGCGTTGTCCAAACAGAAT
Tat	TGCTGGATGTTCGCGTCAATA	CGGCTTCACCTTCATGTTGTC
Ttr	CTGCTGTAGACGTGGCTGTAA	CTTCCAGTACGATTTGGTGTCC
Cps1	TACCCGGAAGCACTTACTGAT	GCCAGCCAGTGGTTATAGTCATT
Cyp1a2	AGTACATCTCCTTAGCCCCAG	GGGTCCGGGTGGATTCTTC
Cyp3a11	CCTGGGTGCTCCTAGCAATC	CAAGGAGAGGCGTTTGACCA
CD133	ACTGGGGCTGTGTGGAAAG	GCATTGAAGGTATCTTGGGTCTC
Epcam	CTGGCGTCTAAATGCTTGGC	CCTTGTCGGTTCTTCGGACTC
Sox9	AGTACCCGCATCTGCACAAC	ACGAAGGGTCTCTTCTCGCT
Alb	CAAGAGTGAGATCGCCCATCG	TTACTTCCTGCACTAATTTGGCA
Actin	ATGCCACAGGATTCCATACCCAAG	CTCTAGACTTCGAGCAGGAGATGG
GAPDH	GGCATGGACTGTGGTCATGAG	TGCACCACCAACTGCTTAGC

**α-SMA**	**CCCAGACATCAGGGAGTAATGG**	**TCTATCGGATACTTCAGCGTCA**
**COLLα1**	**ATCGGTCATGCTCTCTCCAAACCA**	**ACTGCAACATGGAGACAGGTCAGA**
**Desmin**	**GTTTCAGACTTGACTCAGGCAG**	**TCTCGCAGGTGTAGGACTGG**
**Nestin**	**CCCTGAAGTCGAGGAGCTG**	**CTGCTGCACCTCTAAGCGA**
**GFAP**	**TCTCGAATGACTCCTCCACTC**	**AAGCTCCGCCTGGTAGACAT**
**ACTB**	**GCCTCGCTGTCCACCTTCC**	**TGCTGTCACCTTCACCGTTCC**
**H-CD24**	**CTCCTACCCACCGCAGATTTATTC**	**AGAGTGAGACCACGAAGAGAC**
**H-Alb**	**GAGACCAGAGGTTGATGTGATG**	**AGTTCCGGGGCATAAAAGTAAG**
**H-α-SMA**	**AAAAGACAGCTACGTGGGTGA**	**GCCATGTTCTATCGGGTACTTC**
